# Reconstruction of a lncRNA-Associated ceRNA Network in Endothelial Cells under Circumferential Stress

**DOI:** 10.1155/2020/1481937

**Published:** 2020-02-14

**Authors:** Zhuhui Huang, William Adiwignya Winata, Kui Zhang, Yang Zhao, Yang Li, Ning Zhou, Shaoyou Zhou, Wei Fu, Bokang Qiao, Guoqi Li, Yihui Shao, Jubing Zheng, Ran Dong

**Affiliations:** ^1^Department of Cardiac Surgery, Beijing Anzhen Hospital, Capital Medical University, Beijing Institute of Heart, Lung and Blood Vessel Diseases, Beijing 100029, China; ^2^Key Laboratory of Remodeling-Related Cardiovascular Diseases, Capital Medical University, Ministry of Education and Beijing Anzhen Hospital, Beijing Institute of Heart, Lung and Blood Vessel Diseases, Beijing 100029, China

## Abstract

**Background:**

Numerous studies have highlighted that long noncoding RNA (lncRNA) can indirectly regulate the expression of mRNAs by binding to microRNA (miRNA). LncRNA-associated ceRNA networks play a vital role in the initiation and progression of several pathological mechanisms. However, the lncRNA-miRNA-mRNA ceRNA network in endothelial cells under cyclic stretch is seldom studied.

**Methods:**

The miRNA, mRNA, and lncRNA expression profiles of 6 human umbilical vein endothelial cells (HUVECs) under circumferential stress were obtained by next-generation sequencing (NGS). We identified the differential expression of miRNAs, mRNAs, and lncRNAs using the R software package GDCRNATools. Cytoscape was adopted to construct a lncRNA-miRNA-mRNA ceRNA network. In addition, through GO and KEGG pathway annotations, we analyzed gene functions and their related pathways. We also adopted ELISA and TUNEL to investigate the effect of si-NEAT1 on endothelial inflammation and apoptosis.

**Results:**

We recognized a total of 32978 lncRNAs, 1046 miRNAs, and 31958 mRNAs in 6 samples; among them, 155 different expressed lncRNAs, 74 different expressed miRNAs, and 960 different mRNAs were adopted. Based on the established theory, the ceRNA network was composed of 13 lncRNAs, 44 miRNAs, and 115 mRNAs. We constructed and visualized a lncRNA-miRNA-mRNA network, and the top 20 nodes are identified after calculating their degrees. The nodes with most degrees in three kinds of RNAs are hsa-miR-4739, NEAT1, and MAP3K2. Functional analysis showed that different biological processes enriched in biological regulation, response to stimulus and cell communication. Pathway analysis was mainly enriched in longevity regulating, cell cycle, mTOR, and FoxO signaling pathway. Circumferential stress can significantly downregulate NEAT1, and after transducing si-NEAT1 for 24 h, inflammatory cytokine IL-6 and MCP-1 were significantly increased; furthermore, fewer TUNEL-positive cells were found in the si-NEAT1 treated group.

**Conclusions:**

The establishing of a ceRNA network can help further understand the mechanism of vein graft failure. Our data demonstrated that NEAT1 may be a core factor among the mechanical stress factors and that cyclic stress can significantly reduce expression of NEAT1, give rise to inflammation in the early stage of endothelial dysfunction, and promote EC apoptosis, which may play an essential role in vein graft failure.

## 1. Introduction

Coronary Artery Bypass Grafting (CABG) is an economical and effective treatment for most cases of multiple or left main coronary arteries [1]. During the surgery, the patient's autogenous vein is one of the most commonly used conduits for bypass grafting. However, given that vascular smooth muscle cell (VSMC) could give rise to abnormal neointimal hyperplasia of the veins, as many as 50% of CABG operations end up in failure [2]. Jeremy et al. reported that the reasons for vein graft failure involve physical, chemical, and biological factors, including the adhesion of platelets and white blood cells, the change of hemodynamics, activated matrix metalloproteinases and excessive release of platelet-derived growth factor-BB and thrombin, and the superposition effect of atherosclerosis [3–6]. After the vein was transplanted from the venous system to the arterial system, the inflammation and apoptosis of ECs could be induced by changing the mechanical environment, which plays an important role in several pathological processes, including intimal hyperplasia, atherosclerosis, and occlusion [7]. So far, no ideal method has been found to prevent and deal with such adverse events. Wadey et al. observed the different responses between arterial and vein endothelial cells to blood flow and attributed to the different responses to the epigenetic memory of vastly different hemodynamic environments [8].

A plethora of evidence has shown that miRNAs are involved in the pathogenesis of vein graft failure. Previous studies have shown that the expression of miR-21 was significantly increased in various vascular injury models, and the downregulated miR-21 expression serves to improve the intimal thickening of arterial vessels after grafting [9, 10]. MiR-145 exhibits an inhibitory effect on the proliferation, migration, and phenotype transformation of smooth muscle [11]. Ohnaka et al. conducted a nonvirus transfection of miR-145 and observed that the transplanted vein neointimal thickening was suppressed in rabbits [12]. Jan Fiedler et al. observed that higher expression of miR-24 in the myocardial infarction (MI) model promoted endothelial cell apoptosis and that suppressed miR-24 expression using local transfection adenovirus promoted the formation of new veins, thereby improving blood perfusion [13]. MiR-92a was reported to play the part of inhibiting the proliferation and migration of EC cells; the downregulated miR-92a expression using the antagomir method led to improved vascular endothelialization after balloon injury and reduced intimal thickening [14].

LncRNAs could competitively combine with the miRNA response element (MRE) to repress miRNA's regulation of target mRNAs. Such a lncRNA-miRNA-mRNA competing endogenous RNA (ceRNA) network has been demonstrated in several diseases, yet its application in vein graft failure is to be clarified. Therefore, it is essential to detect the ceRNA coregulatory of ECs suffering from cyclic stress by conducting bioinformatic analysis, which is of great help to construct the systematic regulatory network and to explore the correlation between the main biomarkers.

## 2. Materials and Methods

### 2.1. Cyclic Stretch Stimulation

Human umbilical vein endothelial cells (HUVECs, ScienCell Research Laboratories, San Diego, CA, USA) were maintained in endothelial cell medium (ECM, ScienCell Research Laboratories, San Diego, CA, USA) with 10% fetal bovine serum (FBS, ThermoFisher Scientific, Waltham, MA, USA) at 37°C with 5% CO_2_. The ECs were then plated on the collagen-coated plates (Flexcell International Corporation, McKeesport, PA, USA). We adopted a computer-controlled circumferential stress unit (Flexcell 5100, Flexcell International Corporation, McKeesport, PA) to compose cyclic stretch to HUVEC for 24 hours including a condition of cyclic deformation at 60 cycles/min and elongation at 18%. The control group was maintained in the 6-well plate under the same condition but without mechanical stretch.

### 2.2. RNA Isolation

The HUVECs in two groups were harvested after 24 hours. Total RNA was obtained from ECs using TRIzol Reagent (Invitrogen, Carlsbad, CA, USA) according to the manufacturer's instruction. In brief, cells were lysed in the Eppendorf tubes using TRIZOL reagent, and then RNA was separated and precipitated, finally, the total RNA will be dissolved in DEPC-treated water for further experiments. The RNA concentration and purity were checked using NanoDrop 2000 (ThermoFisher Scientific, Waltham, MA, USA) with criteria of OD A260/A280 (>1.8) and A260/A230 (>1.6), Agilent 2100 Bioanalyzer (Agilent Technologies, Santa Clara, CA, USA) was adopted to access the yield and quality by RIN >7.0, and gDNA contamination was evaluated by gel electrophoresis.

### 2.3. RNA Library Preparation and Sequencing

Ribo-Zero Magnetic Gold Kit (Illumina, San Diego, CA, USA) and NEBNext RNA Library Prep Kit (New England Biolabs, Ipswich, MA, USA) were adopted to prepare the whole transcriptome libraries. Quality control and quantification was done by using the BioAnalyzer 2100 system (Kapa Biosystems, Woburn, MA, USA). The resulting libraries were sequenced on a HiSeq2000 instrument (Illumina, San Diego, CA, USA), and we used approximately 1 *μ*g total RNA to prepare an RNA library according to the protocol. Then, we performed the single-end sequencing (50 bp) on Hiseq2500 (Illumina, San Diego, CA, USA) following the vendor's recommended protocol. We used FastQC software to check for potential sequencing issues and contaminants in the raw sequencing reads. Reads with quality scores below 30, adapter sequences, and primers were trimmed. Reads with a length of <60 bp were discarded sequently. We used TopHat 2.0 to align sequence reads to the human genome (GRCh38), and the results were reconstructed with Cufflinks. All transcriptomes were pooled and merged to generate a final transcriptome using Cuffmerge. After the final transcriptome was produced, Cuffdiff was used to estimate the abundance of all transcripts based on the final transcriptome. For mRNA and lncRNA analyses, the RefSeq and Ensembl transcript databases were chosen as the annotation references. We used the Coding Potential Calculator (CPC) [15] to predict transcripts with coding potential. The transcripts that remained were considered reliably expressed lncRNAs. As for miRNAs, reads with a length <10 nt and >34 nt were discarded. The clean reads were aligned against the miRNA precursor of *Homo sapiens* and other species in miRBase 22.1 [16] to identify known miRNAs. The unannotated sequences were mapped to the human genome to analyze their expression and distribution in the genome and then used to predict potential novel miRNA candidates by the Mireap program.

### 2.4. Construction of lncRNA-miRNA-mRNA ceRNA Network

The clean reads of 6 EC samples were imported and analyzed by R 3.6 software after transformation. We adopted a R package of GDCRNATools to analyze the sequencing data. It is a novel R package for integrative analysis of RNA-seq data, and it allows users to conduct ceRNA networks and other routine analyses based on online databases [17]. In short, lncRNA, miRNA, and mRNA expression profiles of fold change (FC) ≥1.5 and *P* value ≤0.05 were retained. To construct the ceRNA network, we predicted miRNA-mRNA and lncRNA-miRNA interactions based on starbase V3.0 [18], miRcode [19], and miRTarBase [20] based on our sequencing data; then, according to ceRNA hypothesis, the miRNAs negatively regulated by lncRNAs and its downregulated target mRNAs were selected, and the common miRNAs interacting with both lncRNAs and mRNAs were seen as an inclusion criteria. After that, the lncRNA-associated ceRNA network was reconstructed and visualized using Cytoscape software V3.5 (San Diego, CA, USA) based on the R output. We used different colors and shapes to represent the three types of RNA, respectively, and all node degrees were calculated simultaneously using the software plugin CytoHubba.

### 2.5. Functional Enrichment Analysis

To understand the potential regulative role of the lncRNA-miRNA-mRNA network, we used the Database for Annotation, Visualization, and Integrated Discovery (DAVID) bioinformatics resources [21] and WEB-based gene set analysis toolkit (Webgestalt) [22]; to enrich the downstream mRNA to molecular functions and pathways, we enriched KEGG pathways using Wedgestalt, and each pathway was listed and ranked by their enrichment ratio. Furthermore, GO analysis was carried out using the DAVID database, molecular functions, and biological processes, and cellular component of the differentially expressed genes were elucidated.

### 2.6. Quantitative Real-Time Polymerase Chain Reaction (qRT-PCR) Validation

To validate the hub gene that we identified in the bioinformatics analysis, qRT-PCR was performed using the riboSCRIPT mRNA/lncRNA qRT-PCR starter kit (RIBO bio, Guangzhou, China) under instructions. Briefly, total RNA was isolated using TRIzol as previously described, then RNA was reverse transcripted to cDNA using random primer and Oligo (dT), and then qPCR was performed using the three-step method. Relative quantification of lncRNA was calculated using the 2-ΔΔCt method and was normalized to GAPDH as a reference. The template sequence for qRT-PCR is NEAT1 5′-GGCAGGTCTAGTTTGGGCAT-3′; 5′-CCTCATCCCTCCCAGTACCA-3′; GAPDH 5′-CATGGCCTTCCGTGTTCCTA-3′; 5′-CGCCTCCTTTTCCTCTCAT-3′.

### 2.7. Enzyme-Linked Immunosorbent Assay (ELISA)

The levels of inflammatory cytokines secreted by endothelial cells transduced with si-NEAT1 (RIBO bio, Guangzhou, China) and control reagents were measured by ELISA. We transduced the si-NEAT1 into endothelial cells after 3-4 passages using Lipofectamine 3000 Reagent (ThermoFisher Scientific, Waltham, MA, USA) according to the established protocol. After that, ELISA was performed on a 96-well plate using ELISA kits (BD Biosciences, San Diego, CA) according to the manufacturer's protocol. We used a spectrophotometer to measure the absorbance at a wavelength of 450 nm. Several common Inflammatory cytokines including IL-6, MCP-1, and ICAM-1 were measured to confirm the effect of downregulating NEAT1 on endothelial inflammatory.

### 2.8. TUNEL and DAPI Staining

To determine the effect on apoptosis after downregulating NEAT1, we performed TUNEL assay on the endothelial cells described above using the In Situ Cell Death Detection kit (Roche, Mannheim, Germany) according to the product's instructions. The cell slide was fixed in 4% paraformaldehyde at room temperature for 20 mins and washed using PBS for 3 times. After that, we used 1% Triton X-100 to increase cell permeability. The fixed cells were incubated with terminal deoxynucleotide transferase recombinant (rTdT)-catalyzed reaction mixture for 30 min at room temperature. Then, we used Streptavidin-FITC to label the apoptosis cells and DAPI to illustrate the nuclei. Apoptotic cells were photographed under a fluorescence microscope at an excitation wavelength of 450 nm and emission wavelength of 515 nm.

### 2.9. Statistical Analysis

We used SPSS version 23.0 and R version 3.6 to analyze the sequencing data, Student's *t*-tests were adopted to compare the difference between two groups, and repeated measures were tested by one-way analysis. *P* < 0.05 was the threshold to be statistically significant. In addition, we use fold changes and Student's *t*-tests to identify specific RNAs in the sequencing results. The ncRNAs and mRNAs with FC ≥ 1.5 and *P* < 0.05 were considered as differentially expressed.

## 3. Results

### 3.1. Altered lncRNA, miRNA, and mRNA Expression in the Endothelial Cells

**Figure 1:**
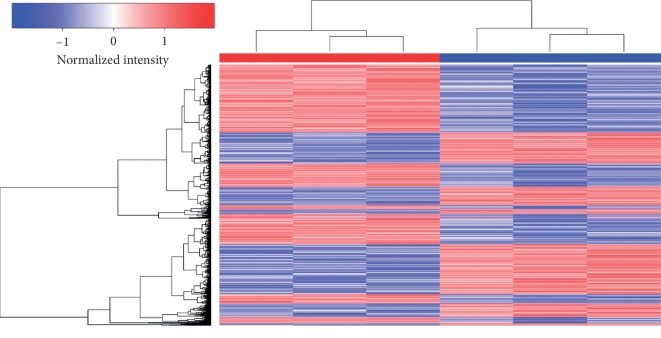
RNA profiling in HUVECs with and without mechanical stretch stimulation. Red boxes represent upregulated genes and blue boxes represent downregulated genes. Each group has 3 replicates.

**Figure 2:**
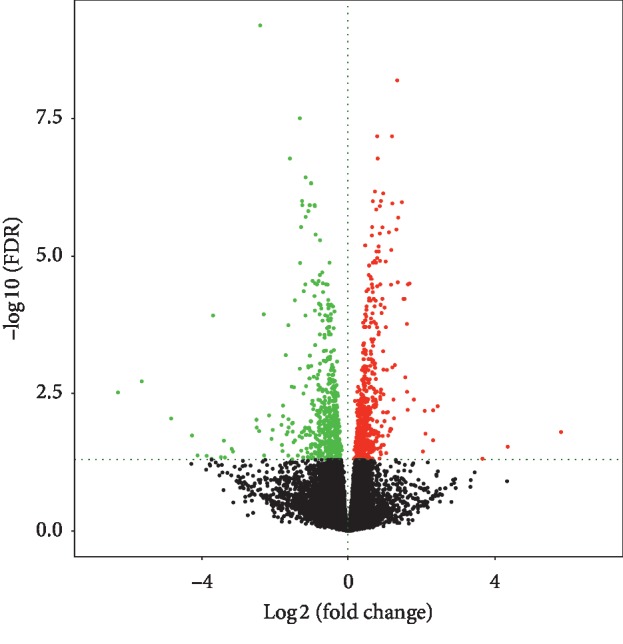
Volcano plot of the differently expressed RNAs. Upregulated genes are marked in light red; downregulated genes are marked in light green. FC ≥ 1.5 and *P* value ≤0.05 were chosen as selective criteria, 155 different expressed lncRNAs, 74 different expressed miRNAs, and 960 different mRNAs were identified.

The expression of lncRNAs, miRNAs, and mRNAs in the endothelial cells under cyclic stretch for 24 hours was profiled using RNA sequencing. After processing the raw data, we identified a total of 32978 lncRNAs, 1046 miRNAs, and 31958 mRNAs in 6 samples of endothelial cells compared with the established databases. We adopted FC ≥ 1.5 and *P* value ≤0.05 as selective criteria; after screening, we got 155 different expressed lncRNAs, 74 different expressed miRNAs, and 960 different mRNAs, among them, 39 lncRNAs, 35 miRNAs, and 568 mRNAs were upregulated, while 116 lncRNAs, 39 miRNAs, and 392 mRNAs were downregulated. The most upregulated were AL031282.2, hsa-miR-151a-5p, and SSUH2; by contrast, the most downregulated were AC092162.2, hsa-miR-100-5p, and RAD21L1. Expression profiles are depicted as heatmap (Figure 1) and dot plot (Figure 2) after normalization; additionally, the top 20 upregulated and downregulated members of each kind of RNAs are listed in Tables 1–3. These results suggest that circumferential stress may vary transcriptional regulation in human endothelial cells.

### 3.2. Reconstruction of lncRNA-Associated ceRNA Network

**Figure 3:**
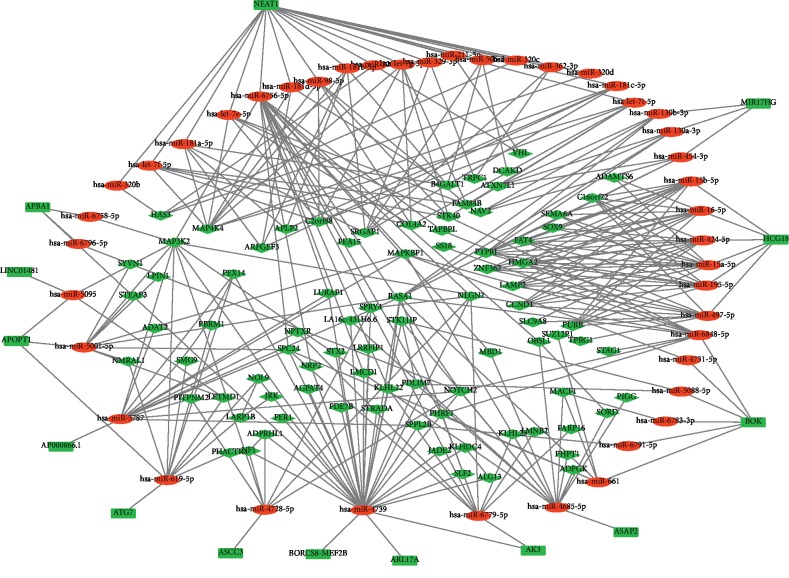
The lncRNA-miRNA-mRNA competing endogenous RNA network in HUVECs stimulated by cyclic stretch. The rectangles indicate lncRNAs in green, ellipses represent miRNAs in red, and diamonds represent mRNAs in light red.

To evaluate whether ceRNA is involved in endothelial cells after stretched, we combined the data acquired in the online database with the data above and constructed a lncRNA-miRNA-mRNA network (Figure 3) based on the ceRNA hypothesis. We identified a total of 13 lncRNAs, 44 miRNAs, and 115 mRNAs as targets in the ceRNA network. In the network, lncRNAs and miRNAs were binding with mRNAs competitively, the cyclic stretch downregulated lncRNAs, thus increased miRNAs and targeted mRNAs indirectly. For example, lncRNA ASCC3 was downregulated, while miR-4728-5p was upregulated, resulting in the decline of Notch2. These data indicated that lncRNA could regulate the expression of mRNA by interacting with miRNA competitively and plays a regulatory role in transcription signals under circumferential stress.

### 3.3. GO and KEGG Analysis of Differentially Expressed mRNAs

**Figure 4:**
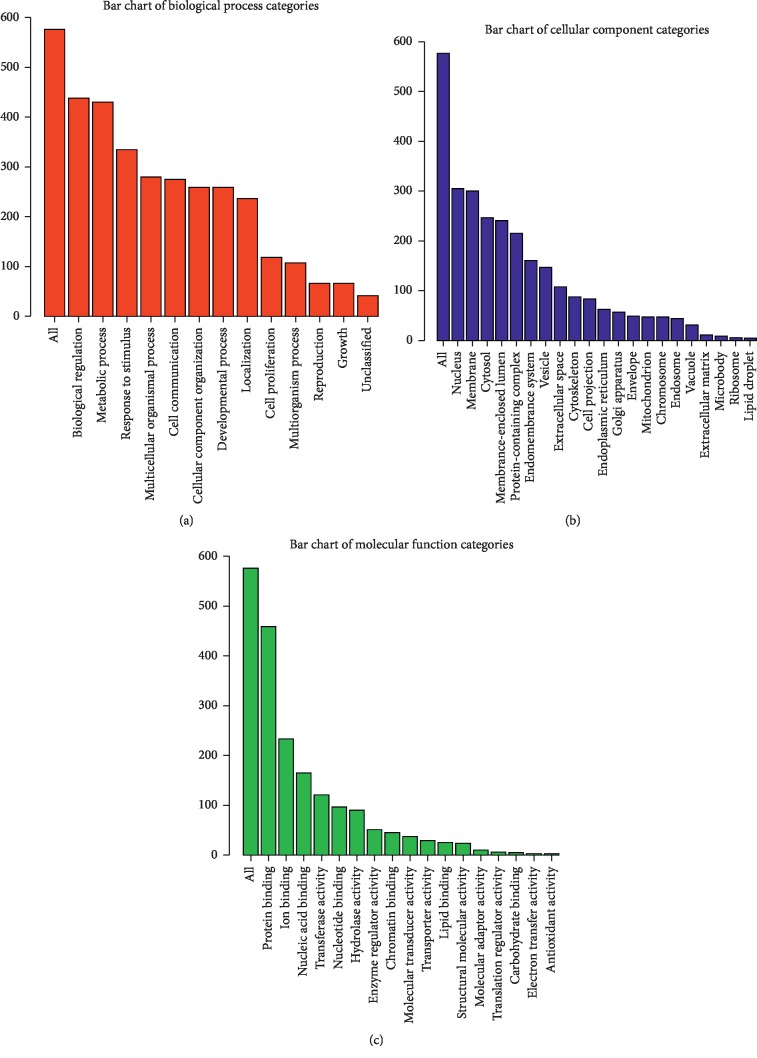
GO enrichment analysis of differentially expressed genes of cyclic stress vein genes (Top 10). (a) Red bar plot of biological process. (b) Blue bar plot of cellular component. (c) Green bar plot of molecular function.

**Figure 5:**
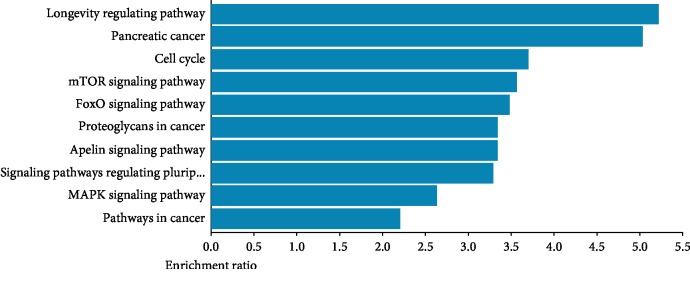
The top 10 enriched KEGG pathways of cyclic stretch mRNAs in the ceRNA networks.

To annotate functions of the mRNAs in the ceRNA network, we performed GO and KEGG enrichment using David tools and WEB-based gene set analysis. GO analysis indicated that most of the genes were involved in biological regulation, metabolic process, and response to stimulus and located in nucleus and membrane; furthermore, the majority of genes were related to protein binding as molecular function (Figure 4), indicating their pivotal role in transcriptional regulation. Meanwhile, KEGG pathway analysis showed that several pathways were associated with the differentially expressed mRNAs including longevity regulating, cell cycle, mTOR signal, FoxO signal, and MAPK signal (Figure 5), which suggests that they may be involved in proliferation and inflammation in endothelial cells.

### 3.4. Identification of lncRNA NEAT1-Associated Subnetwork

**Figure 6:**
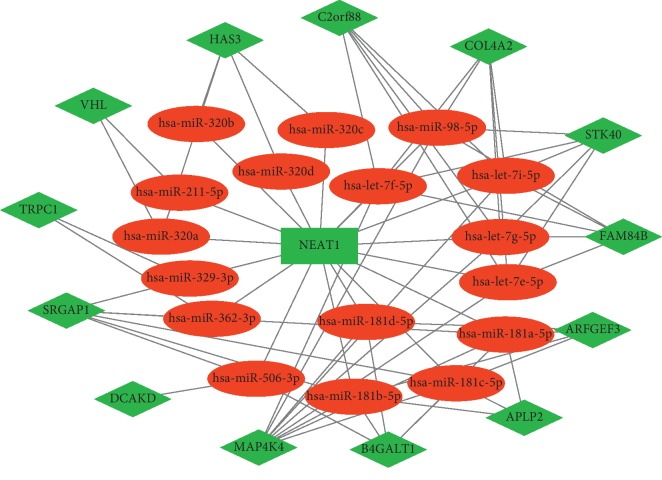
The subnetwork of lncRNA NEAT1 interaction network. The rectangles indicate lncRNAs in green, ellipses represent miRNAs in red, and diamonds represent mRNAs in green.

To identify the hub RNAs and their related networks, we calculated the degrees of each node in the ceRNA network using Cytoscape plugin cytoHubba, and the top 20 nodes were ranked by degrees as shown in Table 4. It showed that lncRNA NEAT1 is among the top-ranked nodes, indicating its important role in transcriptional regulation. Therefore, we extracted a subnetwork of lncRNA NEAT1-miRNA-mRNA out of the ceRNA network, which was composed of 1 lncRNA node, 17 miRNA nodes, 13 mRNA nodes, and 67 edges (Figure 6), and it may be the center of the whole ceRNA network. Several RNAs that had been broadly studied such as miR-let7 cluster and MAP4K4 are involved in the subnetwork, suggesting that the subnetwork may regulate biological processes through various pathways.

### 3.5. Downregulated NEAT1 Validation

**Figure 7:**
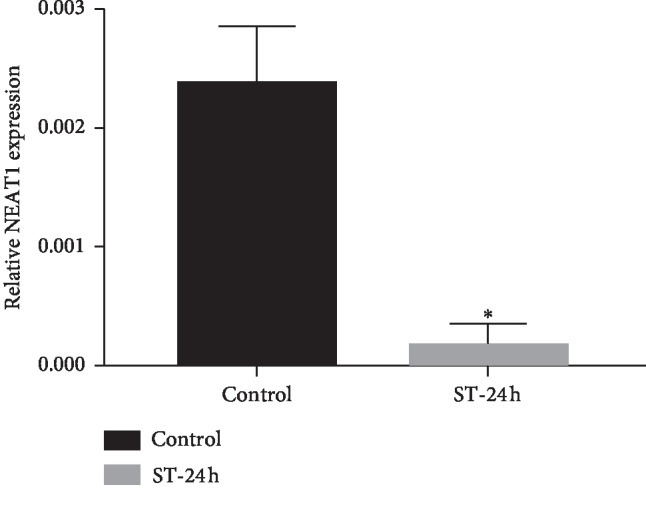
The relative miRNA expression of NEAT1 (normalized to GADPH). NEAT1 level was significantly downregulated in cells of the ST-24 group, in comparison with those of the control group (^*∗*^*P* < 0.05).

In order to validate the expression of NEAT1 in endothelial cells under cyclic stretch, quantitative real-time polymerase chain reaction (qRT-PCR) was performed in the 6 samples above. As shown in Figure 7, lncRNA NEAT1 was significantly downregulated in endothelial cells after being stretched for 24 hours, which was consistent with the RNA-seq data. These results indicated that NEAT1 may play an essential role in the ceRNA network.

### 3.6. Secretion of Inflammatory Cytokines

**Figure 8:**
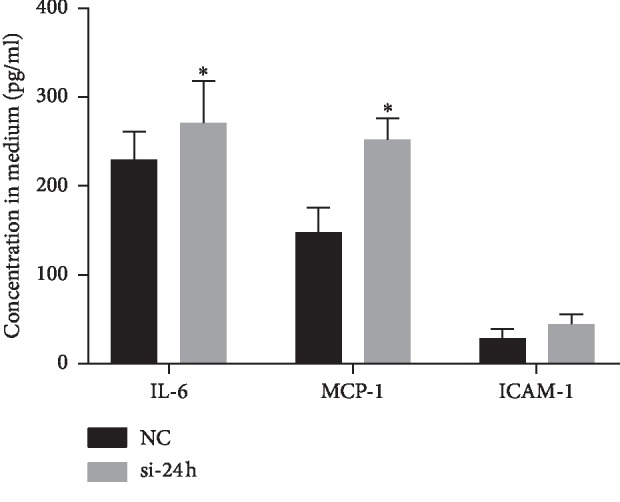
The secretion of inflammatory cytokines measured by ELISA. After si-NEAT1 was transduced into endothelial cells for 24 h, inflammatory cytokines including IL-6 and MCP-1 were significantly increased, comparing to the control group (^*∗*^*P* < 0.05).

To further investigate the effect on inflammatory response after NEAT1 downregulation, we transduced si-NEAT1 into endothelial cells for 24 h and performed ELISA to measure the level of IL-6, MCP-1, and ICAM-1 in the culture medium. After decreasing NEAT1 for 24 h, IL-6 and MCP-1 were significantly increased by about 1 hold (Figure 8), indicating reducing NEAT1 can worsen endothelial inflammatory and may contribute to vein graft failure.

### 3.7. Endothelial Apoptosis Induction

**Figure 9:**
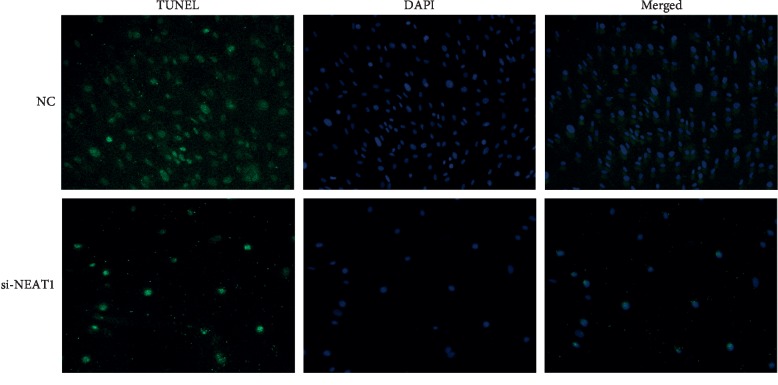
The endothelial cell apoptosis induced by transduction of si-NEAT1. Endothelial cell apoptosis was increased after NEAT1 silencing, and TUNEL-positive cells appeared less in the si-NEAT1 group.

Apart from inflammatory response, we also adopted TUNEL assay to determine the apoptotic effect after downregulating NEAT1. As depicted in Figure 9, fewer TUNEL-positive cells were found in cells treated with si-NEAT1 for 24 h than those with negative control. NEAT1 inhibition leaded to significant endothelial apoptosis, which may result in the aggregation and adhesion of platelet and lead to vein graft failure eventually.

## 4. Discussion

Our results indicate that the lncRNA-associated ceRNA network may function as a transcriptional regulation factor in endothelial cells under cyclic stretch, and we further identified that lncRNA NEAT1 may be a target for further investigation of mechanisms in endothelial cells; by triggering inflammatory response and inducing apoptosis, NEAT1 may play an integral role in vein graft failure and could be a target for RNAi therapy.

Vein graft failure, which was characterized as narrow or occlusion of the saphenous vein grafting from the aorta to the coronary artery, limits the long-term effect of CABG. The velocity of the grafted vein reduced to 80% only 1 year after grafting and then to 40% within 10 years [23], resulting in the long-term patency of vein grafts are merely 60%–65% [24] and causing angina recurrence and revascularization [25]. The mechanotransduction of a grafted vein involves the response to shear stress, cyclic stress, and normal stress. Bondareva reported that oscillatory shear stress (OSS) stimulation for 6 hours could activate the EGR1 and YAP/TAZ complex in human endothelial cells, based on ChIP-seq and luciferase assays [26]. Moreover, Moonen et al. suggested that disturb laminar shear stress (LSS) could aggravate endothelial-to-mesenchymal transition (EndMT), targeting MEK5 signaling, eventually leading to neointimal hyperplasia [27]. Similarly, certain progress has been made in the studies on its effect under circumferential stress. For example, high-cyclic stretch significantly elevated miR-124-3p, downregulating lamin A/C and inducing VSMC apoptosis [28]. Cyclic stretch is an initial factor of vein graft failure; after CABG, the stretch rate of the saphenous vein will be 10–15% due to the pulsating pressure from the aorta [29], which causes over deformation of the endothelium. Endothelial cells, located on the inner layer of the saphenous vein, are sensible to the hemodynamic changes; thus, overstretching them will initiate inflammation and proliferation [30]. As a result, cyclic stretch caused by the aorta triggers endothelial dysfunction, which is a key process of vein graft failure [31]. Furthermore, cyclic stretch induces a large number of growth factors releasing from VSMCs, aggregating endothelial dysfunction, and intimal hyperplasia, which eventually results in vein graft failure [32]. Take together, cyclic stretch stimulation to endothelial cells and its inflammation is the early response of vein graft failure. However, the transcriptional regulation in the endothelial dysfunction remains unclear.

Noncoding RNA is a cluster of RNAs that regulate transcription without expressing into protein, mainly including miRNA, lncRNA, and circRNA [33]. miRNAs are single-stranded and endogenously expressed small noncoding RNAs molecules with lengths of 22 to 24 nucleotides [34]. MiRNA plays a regulatory role in numerous cellular activities, including growth, differentiation, metabolism, apoptosis, and migration [35]. Recently, accumulated studies have shown the role of lncRNAs in various biological processes. Aberrantly expressed lncRNA has been observed in coronary artery disease [36]; furthermore, it was found that lncRNA can regulate mRNA expression by interacting with miRNA, leading to the introduction of ceRNA hypothesis [37]. Further studies have shown that ceRNA genes were mediated by miRNAs which are interacting with increasingly complicated ceRNA networks [38]. The ceRNA network has been found to be involved in the pathogenesis in several diseases; however, very little was found on the association between ceRNA and vein graft failure.

To identify the regulatory role of lncRNA-associated ceRNA network in endothelial dysfunction, we constructed a ceRNA network based on RNA-sequencing data of endothelial cells under cyclic stretch for 24 hours. With FC ≥ 1.5 and *P* value <0.05 threshold, 39 upregulated and 116 downregulated lncRNAs, 35 upregulated and 39 downregulated miRNAs, and 568 upregulated and 392 downregulated mRNAs showed differential expression between two groups. Among them, several genes have been found to be associated with the pathogenesis of vein graft failure. For instance, our previous study found that egr1 was upregulated under cyclic stretch, targeting ICAM-1, leading to vein graft failure [39]. Furthermore, NF-*κ*B is proved to play an integral role in vascular inflammation, and it has been demonstrated that inhibition of NF-*κ*B signaling can reduce the inflammatory response in endothelial cells [40]. On the other hand, activation of the NF-*κ*B pathway is found to impede recovery in the carotid artery injury model [41].

In order to better annotate the biological functions of the downstream mRNAs, we enriched GO and KEGG pathways. Among the most enriched GO terms, biological regulation and response to stimulus have been reported to be involved in endothelial dysfunction. Remarkably enriched KEGG pathways are mTOR, FoxO, and MAPK pathways, and they are known to play an essential role in endothelial dysfunction [42–44], causing inflammation and proliferation of the tunica intima.

Our bioinformatic analysis has also determined that the lncRNA nuclear paraspeckle assembly transcript 1 (NEAT1) is the core of the mechanical stress factor. NEAT1 has been reported as a cancer biomarker [45]. In the current study, the total RNA was extracted after stretching for 24 h and we found that NEAT1 expression was significantly decreased, which was consistent with the result of bioinformatics analysis. NEAT1 is a nuclear-enriched lncRNA located on chromosome 11q13.1 and is considered to promote carcinogenesis and metastasis [46]. The upregulation of NEAT1 has been documented in kidney cancer, ovarian cancer, lung cancer, breast cancer, and glioma cancers, contributing to the accurate prediction of clinical outcomes [47, 48]. Recently, NEAT1 is found to be related to the inflammation response to stimuli [49], regulating the expression of several chemokines and cytokines, including IL-6 and CXCL10, via the MAPK pathway [50]. Our present study indicated that NEAT1 expression was downregulated in endothelial cells after mechanical stretch, subsequently leading to upregulated expression of miRNAs and eventually triggering inflammation response to give rise to vein graft failure. NEAT1 is a potent inflammatory regulator; to the best of our knowledge, our study is the first to report its role in ceRNA.

According to the bioinformatic enrichment and PCR validation, we investigated the endothelial inflammation and apoptosis after NEAT1 was repressed by performing ELISA and TUNEL assays. Similar to the results of previous studies, after transduction of si-NEAT1 for 24 h, inflammatory cytokines were significantly increased in the culture medium. Due to the structural difference between the vein and artery, the endothelial layer of the saphenous vein is prone to cyclic stretch, and the high pressure of the aorta after grafting leads to EC activation and loss, initiating the complex networks and leading to rapid expression of a cascade of adhesion such as MCP-1 and ICAM-1 [51]. Apart from that, endothelial cells are essential parts of vascular homeostasis to maintain a anticoagulant and anti-inflammation environment [52]. TUNEL staining showed that inhibiting NEAT1 can induce endothelial cells apoptosis, which may lead to the exposure of smooth muscle cells, leading to phenotype switch, aggregating intimal hyperplasia, and resulting vein graft failure [53].

Nevertheless, more studies should be carried out to clarify the role of NEAT1 during vein graft failure. Different cyclic stress times and frequencies should be further investigated in future studies in the ceRNA network. In addition, further studies should be made on validation of the downstream genes; moreover, the various reasons to explain the failure of vein graft should be elucidated.

In conclusion, we constructed a lncRNA-associated ceRNA network based on the sequencing data and identified lncRNA NEAT1 is an essential fraction in endothelial dysfunction. We found that cyclic stress in the endothelial cells gives rise to inflammatory response and promotes cell apoptosis by downregulating NEAT1 and its related ncRNAs. Our study provided a novel insight into the transcriptional regulation of vein graft failure.

## Figures and Tables

**Table 1:** Top 40 differently expressed lncRNAs in sequencing analysis.

Gene ID	Gene symbol	*P*	FC	FDR	Regulation
ENSG00000268575	AL031282.2	0.00062	5.79268	0.01580	Up
ENSG00000237813	AC002066.1	0.00341	3.65619	0.04825	Up
ENSG00000234378	AC098828.3	0.00016	2.08850	0.00658	Up
ENSG00000247095	MIR210HG	8.07*E* – 05	1.79213	0.00410	Up
ENSG00000231412	AC005392.2	5.17*E* – 05	1.60687	0.00297	Up
ENSG00000275216	AL161431.1	3.17*E* – 09	1.36434	1.98*E* – 06	Up
ENSG00000268621	IGFL2-AS1	0.00059	1.13821	0.01523	Up
ENSG00000261040	WFDC21P	0.00145	1.96944	0.02708	Up
ENSG00000235852	AC005540.1	7.91*E* – 10	1.89379	9.91*E* – 07	Up
ENSG00000225855	RUSC1-AS1	5.50*E* – 05	1.88656	0.00308	Up
ENSG00000258667	HIF1A-AS2	0.00081	1.84409	0.01852	Up
ENSG00000261780	AC105243.1	1.99*E* – 08	1.83428	8.21*E* – 06	Up
ENSG00000241316	SUCLG2-AS1	1.58*E* – 07	1.79216	3.62*E* – 05	Up
ENSG00000269926	DDIT4-AS1	3.87*E* – 08	1.76033	1.31*E* – 05	Up
ENSG00000228437	LINC02474	1.87*E* – 05	1.71344	0.00141	Up
ENSG00000231721	LINC-PINT	3.13*E* – 07	1.69794	6.14*E* – 05	Up
ENSG00000223901	AP001469.1	0.00123	1.69227	0.02438	Up
ENSG00000167046	AL357033.1	2.17*E* – 07	1.67088	4.62*E* – 05	Up
ENSG00000214049	UCA1	0.00172	1.66915	0.03007	Up
ENSG00000235904	RBMS3-AS3	1.30*E* – 07	1.64632	3.35*E* – 05	Up
ENSG00000230552	AC092162.2	2.81*E* – 05	–5.64846	0.00191	Down
ENSG00000248802	AC078850.2	0.00027	–4.84467	0.00909	Down
ENSG00000234871	LINC01032	0.00285	–3.87282	0.04276	Down
ENSG00000272482	AC254633.1	8.34*E* – 07	–3.69988	0.00012	Down
ENSG00000259437	AC093334.1	0.00308	–3.48527	0.04517	Down
ENSG00000227848	SUCLA2-AS1	0.00314	–3.37469	0.04590	Down
ENSG00000258561	AL359232.1	0.00047	–2.50748	0.01311	Down
ENSG00000225339	AL354740.1	7.71*E* – 07	–2.31612	0.00012	Down
ENSG00000233818	AP000695.2	0.00022	–2.17093	0.00797	Down
ENSG00000255864	AC069208.1	6.51*E* – 06	–1.71555	0.00064	Down
ENSG00000258969	LINC02307	0.00028	–1.64003	0.00936	Down
ENSG00000257114	LINC02450	0.00036	–1.54184	0.01101	Down
ENSG00000227925	LINC01655	3.40*E* – 07	–1.46588	6.51*E* – 05	Down
ENSG00000275119	AC244131.2	0.00281	–1.38628	0.04225	Down
ENSG00000259635	AC100830.1	0.00085	–1.33988	0.01888	Down
ENSG00000225791	TRAM2-AS1	0.00091	–1.27027	0.01999	Down
ENSG00000238042	LINC02257	7.40*E* – 10	–1.26935	9.91*E* – 07	Down
ENSG00000261094	AC007066.2	0.00312	–1.23834	0.04567	Down
ENSG00000257176	AC009318.1	0.00047	–1.18344	0.01311	Down
ENSG00000226370	LINC00375	0.00076	–1.17222	0.01790	Down

**Table 2:** Top 40 differently expressed miRNAs in sequencing analysis.

Gene symbol	*P*	FC	Average expression	Regulation
hsa-miR-151a-5p	0.00830	4.967587	11.843500	Up
hsa-miR-92a-3p	0.00552	4.717289	13.551630	Up
hsa-miR-148b-3p	0.0498	4.493670	12.211111	Up
hsa-miR-21-5p	0.00539	4.433062	16.684564	Up
hsa-let-7i-5p	0.00758	4.169530	11.920628	Up
hsa-miR-30a-5p	0.00624	4.151897	12.066384	Up
hsa-miR-10a-5p	0.00737	3.555440	14.343647	Up
hsa-miR-99b-5p	0.01085	3.496305	14.620504	Up
hsa-let-7f-5p	0.00700	2.995222	11.353220	Up
hsa-miR-222-3p	0.01295	2.850281	10.920330	Up
hsa-miR-4284	0.00777	2.654383	0.863570	Up
hsa-miR-374a-5p	0.01244	2.405461	5.723251	Up
hsa-miR-27b-3p	0.00747	2.125160	11.039688	Up
hsa-miR-18a-5p	0.00708	1.873955	3.569991	Up
hsa-miR-96-5p	0.01107	1.783202	7.315302	Up
hsa-miR-651-5p	0.0435	1.776173	2.523913	Up
hsa-miR-20a-5p	0.01147	1.774804	7.238996	Up
hsa-miR-4521	0.0355	1.773481	7.092690	Up
hsa-miR-210-5p	0.00520	1.502487	2.287306	Up
hsa-miR-10a-3p	0.01273	1.431455	7.732293	Up
hsa-miR-100-5p	0.00749	–4.78274	17.89078	Down
hsa-miR-223-3p	0.00605	–4.61353	0.45163	Down
hsa-miR-144-3p	0.03854	–4.05538	0.32115	Down
hsa-miR-142-5p	0.04626	–3.43605	0.83274	Down
hsa-miR-4772-5p	0.00895	–3.15733	0.86166	Down
hsa-let-7a-5p	0.00921	–2.71715	13.60388	Down
hsa-miR-199a-5p	0.03156	–2.67627	1.37259	Down
hsa-miR-451a	0.04254	–2.37644	2.03836	Down
hsa-miR-3617-5p	0.01273	–2.00633	0.13456	Down
hsa-miR-4443	0.00648	–1.68771	0.85807	Down
hsa-miR-23a-5p	0.00518	–1.54230	1.53357	Down
hsa-miR-3940-3p	0.00762	–1.47825	1.12245	Down
hsa-miR-143-3p	0.00738	–1.47642	4.87572	Down
hsa-miR-191-3p	0.00607	–1.44144	1.31566	Down
hsa-miR-148a-5p	0.00701	–1.33610	1.32972	Down
hsa-miR-7976	0.00962	–1.29245	4.13996	Down
hsa-miR-185-3p	0.01159	–1.27379	1.12633	Down
hsa-miR-27b-5p	0.00107	–1.26108	3.56518	Down
hsa-miR-105-5p	0.01158	–1.25366	1.08368	Down
hsa-miR-576-3p	0.00686	–1.16215	4.20788	Down

**Table 3:** Top 40 differently expressed mRNAs in sequencing analysis.

Gene ID	Gene symbol	*P*	FC	FDR	Regulation
ENSG00000125046	SSUH2	0.00164	4.34497	0.02918	Up
ENSG00000187134	AKR1C1	0.00012	2.44192	0.00543	Up
ENSG00000135373	EHF	0.00016	2.31835	0.00644	Up
ENSG00000231924	PSG1	0.00105	2.31706	0.02229	Up
ENSG00000145358	DDIT4L	0.00070	2.10851	0.01696	Up
ENSG00000182957	SPATA13	0.00221	2.04019	0.03561	Up
ENSG00000147872	PLIN2	1.22*E* – 07	1.67389	3.22*E* – 05	Up
ENSG00000004799	PDK4	1.35*E* – 07	1.63004	3.35*E* – 05	Up
ENSG00000173237	C11orf86	0.00016	1.62141	0.00633	Up
ENSG00000100292	HMOX1	1.34*E* – 06	1.60182	0.00017	Up
ENSG00000178150	ZNF114	2.22*E* – 05	1.55896	0.00162	Up
ENSG00000109846	CRYAB	3.12*E* – 07	1.54027	6.14*E* – 05	Up
ENSG00000114268	PFKFB4	3.11*E* – 07	1.50759	6.14*E* – 05	Up
ENSG00000087086	FTL	9.60*E* – 10	1.46587	1.04*E* – 06	Up
ENSG00000120738	EGR1	1.11*E* – 07	1.35264	3.07*E* – 05	Up
ENSG00000167996	FTH1	8.01*E* – 13	1.33776	6.52*E* – 09	Up
ENSG00000163347	CLDN1	6.03*E* – 09	1.31604	3.27*E* – 06	Up
ENSG00000132196	HSD17B7	0.00027	1.24930	0.00902	Up
ENSG00000128510	CPA4	8.09*E* – 05	1.23659	0.00410	Up
ENSG00000104419	NDRG1	1.08*E* – 09	1.20327	1.10*E* – 06	Up
ENSG00000244588	RAD21L1	5.43*E* – 05	–6.29595	0.00305	Down
ENSG00000268434	AC011530.1	0.00276	–4.12341	0.04191	Down
ENSG00000198049	AVPR1B	0.00341	–3.74115	0.04818	Down
ENSG00000269955	C7orf55	0.00108	–3.40469	0.02263	Down
ENSG00000140807	NKD1	0.00221	–3.15605	0.03561	Down
ENSG00000112139	MDGA1	0.00029	–2.51469	0.00953	Down
ENSG00000118557	PMFBP1	0.00058	–2.43974	0.01516	Down
ENSG00000162496	DHRS3	4.02*E* – 14	–2.41244	0.00000	Down
ENSG00000141469	SLC14A1	0.00097	–2.10327	0.02102	Down
ENSG00000102174	PHEX	0.00054	–2.06964	0.01443	Down
ENSG00000214279	SCART1	0.00039	–1.83248	0.01167	Down
ENSG00000065320	NTN1	0.00145	–1.81924	0.02707	Down
ENSG00000260851	AC010542.3	0.00214	–1.80247	0.03497	Down
ENSG00000273167	AL359736.1	0.00024	–1.78956	0.00822	Down
ENSG00000175093	SPSB4	0.00012	–1.78920	0.00528	Down
ENSG00000054219	LY75	0.00072	–1.68978	0.01736	Down
ENSG00000171189	GRIK1	0.00063	–1.66405	0.01580	Down
ENSG00000104728	ARHGEF10	0.00280	–1.64612	0.04222	Down
ENSG00000134955	SLC37A2	1.42*E* – 06	–1.64513	0.00018	Down
ENSG00000135205	CCDC146	0.00046	–1.63602	0.01302	Down

**Table 4:** The list of differentially expressed genes (node degree >5).

Number	Gene type	Gene symbol	Degree
1	miRNA	hsa-miR-4739	35
2	lncRNA	NEAT1	18
3	miRNA	hsa-miR-6756-5p	17
4	miRNA	hsa-miR-4685-5p	16
5	miRNA	hsa-miR-5787	14
6	miRNA	hsa-miR-15a-5p	12
7	miRNA	hsa-miR-619-5p	12
8	mRNA	MAP3K2	11
9	miRNA	hsa-miR-424-5p	11
10	miRNA	hsa-miR-15b-5p	11
11	miRNA	hsa-miR-497-5p	11
12	miRNA	hsa-miR-16-5p	11
13	miRNA	hsa-miR-6779-5p	11
14	miRNA	hsa-miR-6848-5p	10
15	mRNA	RASA1	10
16	miRNA	hsa-miR-195-5p	10
17	miRNA	hsa-miR-5001-5p	10
18	mRNA	MAP4K4	9
19	mRNA	PTPRJ	9
20	miRNA	hsa-miR-4728-5p	8

## Data Availability

The data used to support the findings of this study are available from the corresponding author upon request.
